# Genome Sequencing of *Cladobotryum protrusum* Provides Insights into the Evolution and Pathogenic Mechanisms of the Cobweb Disease Pathogen on Cultivated Mushroom

**DOI:** 10.3390/genes10020124

**Published:** 2019-02-08

**Authors:** Frederick Leo Sossah, Zhenghui Liu, Chentao Yang, Benjamin Azu Okorley, Lei Sun, Yongping Fu, Yu Li

**Affiliations:** 1 Engineering Research Center of Chinese Ministry of Education for Edible and Medicinal Fungi, Jilin Agricultural University, Changchun 130118, China; flsossah@gmail.com (F.L.S.); bazu_okorley@st.ug.edu.gh (B.A.O.); sunlei@jlau.edu.cn (L.S.); 2 Department of Plant Protection, Shenyang Agricultural University, Shenyang 110866, China; zhenghuiliu1212@126.com; 3 BGI-Shenzhen, Shenzhen 518083, China; China National GeneBank, BGI-Shenzhen, Shenzhen 518083, China; yangchentao@genomics.cn

**Keywords:** *Cladobotryum protrusum*, mycoparasite, cobweb disease, *de novo* assembly, SMRT sequencing

## Abstract

*Cladobotryum protrusum* is one of the mycoparasites that cause cobweb disease on cultivated edible mushrooms. However, the molecular mechanisms of evolution and pathogenesis of *C. protrusum* on mushrooms are largely unknown. Here, we report a high-quality genome sequence of *C. protrusum* using the single-molecule, real-time sequencing platform of PacBio and perform a comparative analysis with closely related fungi in the family Hypocreaceae. The *C. protrusum* genome, the first complete genome to be sequenced in the genus *Cladobotryum*, is 39.09 Mb long, with an N50 of 4.97 Mb, encoding 11,003 proteins. The phylogenomic analysis confirmed its inclusion in Hypocreaceae, with its evolutionary divergence time estimated to be ~170.1 million years ago. The genome encodes a large and diverse set of genes involved in secreted peptidases, carbohydrate-active enzymes, cytochrome P450 enzymes, pathogen–host interactions, mycotoxins, and pigments. Moreover, *C. protrusum* harbors arrays of genes with the potential to produce bioactive secondary metabolites and stress response-related proteins that are significant for adaptation to hostile environments. Knowledge of the genome will foster a better understanding of the biology of *C. protrusum* and mycoparasitism in general, as well as help with the development of effective disease control strategies to minimize economic losses from cobweb disease in cultivated edible mushrooms.

## 1. Introduction

As the commercial cultivation of edible mushrooms continuously expands worldwide, the occurrence of diseases caused by fungal pathogens is also increasing, seriously affecting mushroom quality and yield [1]. Cobweb disease is one of the most important limiting factors in mushroom production [2]. Members of the genus *Cladobotryum*, belonging to the kingdom Fungi, division Ascomycota, class Sordariomycetes, order Hypocreales, and family Hypocreaceae, are causal agents of cobweb disease on a number of economically important mushroom crops, such as *Agaricus bisporus*, *Flammulina velutipes*, *Pleurotus ostreatus*, *P. eryngii*, *Hypsizygus marmoreus*, and *Ganoderma lucidum* [3,4,5,6,7,8]. The species *C. dendroides*, *C. mycophilum*, *C. protrusum,* and *C. varium* are pathogens that frequently cause cobweb disease in commercial mushroom farms. The characteristic symptom of cobweb disease is the abundance of coarse mycelium [9], which covers the affected mushrooms with numerous spores and spreads rapidly in commercial growth rooms, leading to serious economic losses worldwide [5,10,11]. 

Among the *Cladobotryum* genus, *C. protrusum* is an important member, as it causes cobweb disease on edible mushrooms, such as *Coprinus comatus*, *Agaricus bisporus*, and *P. ostreatus*, and has the widest distribution [12,13]. The taxonomy, classification, incidence, distribution, and host range of *C. protrusum* have been well studied [12,13]. The phylogenetic placement of *C. protrusum* within the genus *Cladobotryum* has been inferred from the internal transcribed spacer (ITS), translation-elongation factor 1-alpha, and DNA-directed RNA polymerase II subunit *RPB1* and *RPB2* genes [13]. Beyond this study, no genetic resources of *C. protrusum* have been developed. Specifically, the infection mechanism of mycoparasitism is largely unknown, and, in particular, the genes related to pathogenicity, virulence, cell wall degrading enzymes, and secondary metabolites (SMs) are undetermined. Therefore, the sequenced genome could serve as an important genetic resource for further evolutionary studies of the *Cladobotryum* genus and facilitate the elucidation of the pathogenic mechanisms of *C. protrusum.*

The *Cladobotryum* genus comprises at least 66 species [12], and genome sequencing has not been performed on any of them. The development of next-generation sequencing technologies, such as Illumina, 454 sequencing platforms, and the single-molecule real-time (SMRT, PacBio) sequencing platform, has led to the sequencing of many fungal genomes [13]. PacBio sequencing technology offers increased read lengths, unbiased genome coverage, and simultaneous identification of mutation sites [14,15,16]. Sequenced genomes provide data that allow us to gain insights into fungal growth, evolution, and host–pathogen interactions as well as identifying genes related to pathogenicity and the synthesis of SMs of economic importance [17].

In this study, we report the de novo genome sequencing of *C. protrusum* generated using the SMRT sequencing platform, which is the first genome to be sequenced in the *Cladobotryum* genus. We aim (1) to present a high-quality reference genome for *C*. *protrusum* and an analysis of genes related to its pathogenicity and mycoparasitism and (2) to conduct a comparative genome analysis using other sequenced genomes from species within the Hypocreaceae family. The genome assembly will further expand genomic datasets for comparative genomics of species in the Hypocreaceae family and mycoparasites in general. This study will promote the understanding of the biology of *C. protrusum* and the development of effective strategies for controlling cobweb disease.

## 2. Materials and Methods

### 2.1. Fungal Strain and Genomic DNA Extraction

The *C. protrusum* strain used in this study was a single spore isolate collected from the Institute of Applied Mycology, Huazhong Agricultural University, Wuhan, Hubei, China, which was maintained on potato dextrose agar (Difco™, Fisher Scientific, Pittsburgh, PA, USA). The fungal strain was isolated from *C. comatus* from a mushroom farm in Wuhan [18]. The identity of the fungus was confirmed by morphological characteristics, PCR amplification, and sequencing of the ITS gene sequence of the genomic DNA and a BLAST search on the GenBank database. Mycelium plugs of pure isolates were cultured on PDA overlaid with cellophane membrane and incubated at 25 °C for three days under a 12 h white light photoperiod. Genomic DNA was extracted from mycelia using the CWBiotech Plant DNA extraction kit (CWBiotech Corporation, Beijing China) following the manufacturer’s instructions. The quality of DNA was verified with 1% agarose gel electrophoresis and visualization with Gel Doc™ XR+ (Bio-Rad, USA). DNA quantification was done using a Qubit 4.0 fluorometer (Invitrogen, Carlsbad, CA, USA).

### 2.2. Genome Sequencing and Assembly

A genomic DNA library was constructed using a SMRTbell Template Prep kit (Pacific Biosciences, CA, USA) in accordance with the manufacturer’s protocol. A BluePippin device (Sage Science, Inc., Beverly, MA, USA) was used to select 20 kb insert size fragments for the SMRTbell Template library. Quality inspection and quantification of the size-selected library were done using an Agilent 2100 Bioanalyzer (Agilent Technologies, Santa Clara, CA, USA) and Qubit 4.0 fluorometer (Invitrogen, Carlsbad, CA, USA). Prepared whole-genome libraries were sequenced on a PacBio Sequel sequencer (Pacific Biosciences, Menlo Park, CA, USA) with one SMRT cell at the Engineering Research Center of the Chinese Ministry of Education for Edible and Medicinal Fungi, Jilin Agricultural University, Changchun, China. The genome was assembled using SMARTdenovo as described below, in accordance with www.github.com/smartdenovo. The completeness of the assembled genome was evaluated using the Core Eukaryotic Genes Mapping Approach (CEGMA) [19] and Benchmarking Universal Single-Copy Orthologs (BUSCO v3) [20,21] with conserved orthologous gene profiles for fungi.

### 2.3. Gene Prediction and Annotations

The assembled genome was annotated using a homology-based method and de novo prediction methods. Genewise [22] was used for the homology search using the proteomes of *Fusarium redolens*, *Fusarium oxysporum* FOX64, *Neurospora crassa*, and *Trichoderma atroviride* (available from http://www.uniprot.org/; release 2012_07) as training sets. De novo prediction of the protein-coding genes was done using Augustus v2.7 [23], GlimmerHMM v3.02 [24], Genscan v1.0 [25], and SNAP v 2006-07-28 [26]. GLEAN was used to integrate all of the gene models to produce a non-redundant reference gene set (http://glean-gene.sourceforge.net/) [27]. The repeat sequences were identified and masked using RepeatModeler v1.0.7 and RepeatMasker v4.0.5. Tandem repeats were identified by the Tandem Repeats Finder (TRF) v4.04 (http://tandem.bu.edu/trf/trf.html) [28] by searching the repeat sequences against the Repbase database (http://www.girinst.org/repbase/) [29]. Transfer RNAs were predicted using tRNAscan-SE 1.3.1 [30], whereas rRNAs and noncoding RNAs were identified using RNAmmer 1.2 [31] and the Rfam database [32]. The predicted-coding sequences were functionally annotated by BLASTP (e-value cutoff of 1×10^−5^) query against several protein databases such as the National Center for Biotechnology Information (NCBI) non-redundant (nr), Cluster of Orthologous Groups (COG) [33], the Gene Ontology (GO) database [34], the Kyoto Encyclopedia of Genes and Genomes (KEGG) database [35], the SwissProt database [36,37,38], the TrEMBL databases [38], and the InterPro Protein Families Database (IPR including Pfam database) [39]. The mating-type genes for *C. protrusum* were determined by BLAST (tBLASTx e-value 1×10^−30^) similarity searches using mating-type genes and flanking gene sequences from the order Hypocreales retrieved from NCBI database. The gene structure was drawn using the software package illustrator of biological sequences version 1.0 (http://ibs.biocuckoo.org/) [40].

### 2.4. Orthologous Gene Families and Phylogenomic Analysis

An all-vs.-all BLASTP with an e-value cutoff of 1×10^−5^ was used to compare the proteins of ten species including *C. protrusum* (CPR), *Clonostachys rosea* (CR), *Fusarium solani* (FS), *Magnaporthe grisea* (MG), *Metarhizium acridum* (MA), *N. crassa* (NA), *Tolypocladium inflatum* (TI), *T. longibrachiatum* (TL), *T. reesei* (TR), and *T*. *virens* (TV) (Supplementary Table S1). The BLAST results were clustered by a MATLAB implementation of the Markov Clustering (MCL) algorithm (MMCL) [41] to identify orthologous groups using OrthoMCL (v. 2.0.9) [42]. Multiple sequence alignment was performed on the proteins of single-copy orthologs identified using MUSCLE [43]. The phylogenetic tree was used for maximum-likelihood (ML) analysis by RAxML-8.0.26 [44] using the LG+I+G+F amino acid substitution matrix model selected by ProtTest (v. 3.4) [45] with 1000 bootstrap replicates.

The species divergence times were inferred with the MCMCTree included in the PAML v4.7a software package [46] with r8s v1.81 (http://loco.biosci.arizona.edu/r8s/) [47]. The divergence times were estimated using the approximate method with fossil calibrations from http://www.timetree.org [48]. The expansion of the orthologous gene families and contraction across organisms was calculated by Computational Analysis of Gene Family Evolution (CAFE) (v. 3) [49] with a stochastic birth and death model using a lambda value of 0.314, a *p*-value of 0.01, and 1000 random samples [50]. The genes under selection pressure were identified by calculating the dN/dS ratio between the species in the phylogenetic tree (*p* ≤ 0.01) using the Codeml program PAML [46]. 

Furthermore, OrthoVenn [51] was used for genome-wide identification, comparison, and visualization of unique and shared orthologous gene clusters for *C. protrusum*, *Escovopsis weberi*, *T. reesei*, and *T. virens*. In addition, the proteomes of *C. protrusum*, *E. weberi*, *T. reesei*, *T. virens*, *M. grisea,* and *Aspergillus nidulans* were clustered into orthologous groups using OrthoFinder [52]. The multiple sequence alignments of the single-copy orthologs was used for phylogenetic analysis using the Neighbor-Joining method, which was conducted in MEGAX, to validate the relationships among *C. protrusum* and the other three fungi in the family Hypocreaceae [53].

### 2.5. Secretory Protein Analysis and Pathogenicity-Related Genes

Secretory proteins were predicted using SignalP 3.0 [54]. Transmembrane proteins were predicted by TMHMM [55]. Protein localization signals, excluding those related to the plastid location, were identified using TargetP [56]. Glycosylphosphatidylinositol (GPI)-anchored proteins were predicted using the PredGPI server [57]. Transporters were analyzed through local BLASTP against the Transporter Classification Database (TCdb) with a cutoff e-value of 1×10^−40^ [58]. Proteases were identified with BLASTP (e-value 1×10^−30^) by searching the secretory proteins against the MEROPS database [59]. Cytochrome P450s were classified based on BLASTP alignment against the P450 database with a cutoff e-value of 1×10^−20^ (http://drnelson.uthsc.edu/CytochromeP450.html) [60]. To identify virulence-associated genes, BLASTP (with a cutoff e-value of 1×10^−5^) searches of the *C. protrusum* genome were performed against protein sequences in the pathogen–host interaction database (PHI) (version 3.2, http://www.phi-base.org/) [61] and the database of fungal virulence factors (DFVF) [62]. Carbohydrate-active enzymes (CAZymes) were determined using the dbCAN 2 meta server [63]. SMs were annotated using the antiSMASH (http://antismash.secondarymetabolites.org) fungiSMASH option [64] database and NaPDoS (http://napdos.ucsd.edu) [65].

## 3. Results

### 3.1. Genome Sequencing and Assembly of C. protrusum

The genome of *C. protrusum* was sequenced using the PacBio SMRT Sequel platform. In total, 587,476 sub-reads were generated, representing 6.23 Gb of sequence data at 160 X coverage. The de novo assembly of the *C. protrusum* genome yielded ~39.09 Mb, consisting of 18 scaffolds (Table 1) with a scaffold N50 length of 4.97 Mbp and a scaffold N90 length of 1.93 Mbp. The guanine-cytosine content (GC-content) of the *C. protrusum* genome was 47.84%. CEGMA [19] analysis revealed that 97.58% of the core eukaryotic genes were contained in the assembly (242 out of 248 core eukaryotic genes), while the BUSCO [21] assessment results showed that 99.7% (289 out of 290 genes) of genes were covered by the assembled genome containing 99%, 0.7%, and 0.3% of complete, duplicated, and missing BUSCOs [21], respectively. Therefore, the CEGMA [19] and BUSCO [21] results indicate that the assembled genome for *C. protrusum* was of a high quality. The genome of *C. protrusum* has been deposited into the NCBI database with the accession number RZGP00000000.

### 3.2. Genome Annotation

**Figure 1:**
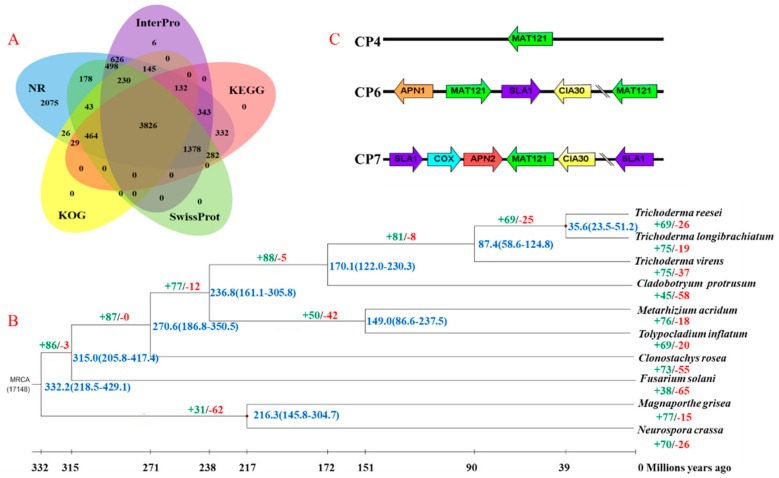
Annotation, phylogenetic and divergence time tree, and mating-type gene structure of the *C. protrusum* genome assembly. (**A**) Functional annotation of the protein-coding genes in the *C. protrusum* genome. (**B**) Phylogenetic and divergence time tree of *C. protrusum* and other nine fungal species. The phylogenetic tree was generated from 3279 single-copy orthologs using the maximum-likelihood method. The divergence time range is shown in blue text, the numbers in green/ red show the proportion of expanded/contracted gene families in each fungal species. (**C**) Schematic representation of the structure of mating-type loci (*MAT 1-2-1*) in *C. protrusum*. The arrows represent the orientation of the *MAT1-2* genes *SLA*, *APN*, *CIA30*, and *COX*.

Genome annotation based on de novo prediction and a homology-based search identified 11,003 protein-coding genes with an average sequence length of 1723.49 bp (Table 1). Overall, 10,623 (96.55%) of the predicted genes had known homologs in at least one functional protein database. Among these proteins, 10,607 (96.40%) were similar to the sequences in the NCBI nr database, 6899 (62.70%) homologs were similar to sequences in Swiss-Prot, 6786 (61.67%) were mapped to KEGG, 4895 (44.49%) were classified in COG, 10,587 (96.22%) were classified in TrEMBL, 7184 (65.29%) were classified in InterPro, and 5332 (48.46%) were assigned to GO terms (Figure 1A). In addition, the proportion of transposable elements (TEs) in *C. protrusum* was estimated to be 2.59% based on combined homology-based and de novo approaches (Table 1). The TEs were randomly distributed across all chromosomes, and the Class I TEs (retrotransposons) (1.34%) were more abundant than the Class II TEs (DNA transposons) (0.48%). The unknown TEs represented 1.24% of the total, and the most abundant characterized TEs in the *C. protrusum* genome were long terminal repeat (LTR) retrotransposons, which accounted for 0.67% of the genome (Supplementary Table S2). A total of 242 tRNAs and 225 rRNAs of the non-coding RNA species were identified in the *C. protrusum* genome. We also predicted 97 miRNAs and 22 snRNAs.

### 3.3. Identification of Mating-Type Idiomorphs in C. protrusum

*MAT1-2* mating-type idiomorphs were identified in the genome of *C. protrusum*, whereas the *MAT1-1* idiomorph (1α domains) was not. The *MAT1-2* idiomorphs were located on different scaffolds (utg37, utg67 (two genes), and utg83) and were distant from each other (Figure 1C). This result suggests that *C. protrusum* has tetrapolar nuclei and confirms our previous observations under a microscope, which showed four nuclei. The cytoskeleton assembly control protein, AP endonuclease, cytochrome C oxidase subunit VIa, and complex I intermediate-associated protein 30 kDa genes were found to flank the *MAT1-2* idiomorph on utg67 and utg83.

### 3.4. Genome Evolution and Phylogenomic Analysis of C. protrusum

A total of 122,201 genes from ten species, including CPR, CR, FS, MG, MA, NA, TI, TL, TR, and TV, were clustered into 11,976 orthogroups using OrthoMCL. Among them, 4761 (39.75%) gene families were shared among all ten species, while 3279 (27.38%) were single-copy orthologous genes. A total of 862 (7.20%) gene families were found to be unique to *C. protrusum* when compared to the other genomes. The single-copy orthologous genes were used for the phylogenetic analysis of the above-mentioned ten species, which was conducted to determine the relationship between *C. protrusum* and other important members in the class Sordariomycetes (Figure 1B). The phylogenetic analysis resolved the ten species into three orders—Hypocreales, Magnaporthales, and Sordariales—with five families in Hypocreales clustered in a different node with NA and MG under separate nodes as outgroups. The orders Hypocreales, Magnaporthales, and Sordariales diverged from the most recent common ancestor (MRCA) 332.2 million years ago (MYA). *C. protrusum* clustered with *Trichoderma* spp. and was separated into different clades based on the genus. The phylogenetic tree confirmed that *C. protrusum* belongs to the Hypocreaceae family and diverged from the genus *Trichoderma* about 170.1 MYA. The results indicate that *C. protrusum* and *Trichoderma* spp. are distantly related to each other at the family level.

The expansion and contraction of the analysis of gene families showed that 88 (2.68%) gene families expanded and five gene families contracted in the family Hypocreaceae based on the 3279 shared gene families from the phylogenetic tree (Figure 1B). Furthermore, we found that *C. protrusum* gained 45 gene families and lost 58 (1.77%) gene families. Except for *C. protrusum* and *F. solani,* the gain of gene families occurred more often than gene loss in the species analyzed. The expanded gene families contain 245 (7.47%) genes (Supplementary Table S3) with several genes related to metabolism, transcription, proteins with binding functions, signal transduction mechanisms, cell rescue and defense protein transport, and synthesis of SMs. Moreover, the gene families exhibiting the largest expansions in *C. protrusum* include zinc-binding dehydrogenase transcription factors, major facilitator superfamily (MFS) transporters, alcohol dehydrogenases, ankyrin-repeat proteins, ATP-binding cassette (ABC) transporters, polyketide synthases (PKSs), and cytochrome P450 monooxygenases (CYP). Interestingly, genes involved in the mediation and regulation of SM synthesis were the most abundant and included genes such as acyl transferase domain, AMP-binding enzyme, beta-ketoacyl synthase, C-terminal domain, condensation domain, insecticide toxin TCdb middle, methyltransferase domain, polyketide synthase dehydratase, and keto-reductase domain. We also found the vegetative incompatibility or heterokaryon incompatibility protein (HET) in the *C. protrusum* genome. 

We identified a total of 3196 genes under selection pressure among the species. Out of these 3196 genes, 14.05% (449 genes) and 24.50% (783 genes) were under positive selection in *C. protrusum* at *p*-values of *p* < 0.01 and *p* < 0.05 respectively (false discovery rate, FDR < 0.1). The positive selection genes (PSGs) were functionally annotated in the GO, KEGG, Pfam, and SwissProt databases (Supplementary Table S4, and Figures S1 and S2). The most abundant GO terms for PSGs were related to cellular component (Figure S1), and, of these, cell (154), cell part (152), and organelle (120) were the three most common GO terms. The PSGs were subsequently analyzed for enrichment in GO categories and KEGG pathways. The analysis revealed 64 enriched metabolic KEGG pathways (Supplementary Table S5), whereas no GO terms were enriched for the PSGs. Further analysis of the PSGs showed that 52 (11.58%) genes are involved in PHI and the majority of the PSGs that played roles in mycoparasitism, include CYP, MFS, SMs, the glycosyl hydrolases family, peptidases, lipases, the subtilase family, and transcription factors.

### 3.5. The Orthologous Genes of C. protrusum and Three Other Fungi in the Hypocreaceae Family

**Figure 2:**
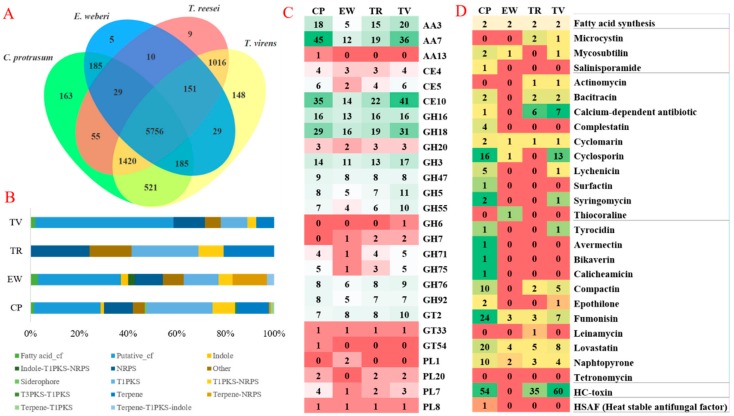
Comparative genomic analysis, carbohydrate-active enzymes (CAZymes) and secondary metabolites (SMs) of *C. protrusum* and three other fungi in the Hypocreaceae family. (**A**) Comparison of the protein-coding genes of *C. protrusum* with those of other Hypocreaceae with different lifestyle *E. weberi* (27.20 Mb, 6870 genes), TR (33.39Mb, 9115 genes) and TV (39.02Mb, 12,406 genes) based on orthology analysis. (**B**) The number of antiSMASH SMs of *C. protrusum* and EW, *T. reesei* (TR), and *T*. *virens* (TV). (**C**) Abundance of CAZyme modules in *C. protrusum* and EW, TR, and TV. (**D**) The number of NaPDoS SMs of *C. protrusum* and EW, TR, and TV.

A phylogenetic tree was constructed using the single-copy orthologs from the clustered proteomes of *C. protrusum*, *E. weberi*, *T. reesei*, *T. virens, M. grisea,* and *A. nidulans*. The tree (Figure S3) depicts the relationships among *C. protrusum* and the other three fungi in the family Hypocreaceae. *M. grisea* and *A. nidulans* were used as outgroups in the tree. We used OrthoVenn [51] to cluster orthologous genes and compared the proteomes of *C. protrusum* against *E. weberi*, *T. reesei*, and *T. virens*, (Supplementary Table S1) which belong to the same family, i.e., Hypocreaceae. The species formed 9682 orthologous clusters and 9357 (96.64%) clusters for at least two species. Among them, 5756 (59.45%) orthologous clusters were shared among all four species (Figure 2A). The top three Swiss-Prot annotations among the core shared orthologous proteins include the ATP-binding cassette transporter (13 proteins), the F-box protein (11 proteins), and the Leptomycin B resistance protein (9 proteins) (Supplementary Table S6). The unique orthologous clusters are 168 (1.74%), 5 (0.05%), 9 (0.09%), and 148 (1.53%) for *C. protrusum*, *E. weberi*, *T. reesei*, and *T. virens*, respectively. Similarly, *C. protrusum* had the most enriched GO categories (23) followed by *T. virens* (9), while *E. weberi* and *T. reesei* had no known annotations or GO enrichment (Supplementary Table S7). Most of the unique genes to *C. protrusum* are related to SM biosynthesis. There were 521 (5.38%) gene clusters shared between *C. protrusum* and *T. virens*, 185 (1.91%) for *C. protrusum* and *E. weberi*, and 55 (0.57%) for *C. protrusum* and *T. reesei*. The highest gene cluster shared between any two species with the most enriched GO categories was observed for *T. reesei* and *T. virens*. This could be because they belong to the same genus. The gene clusters of the enriched GO for *T. reesei* and *T. virens* as well as *C. protrusum* and *T. reesei* were related to transport and cell enzyme degradation. The gene clusters of the enriched GO for *C. protrusum* and *E. weberi* were SMs, especially genes related to toxins and pigmentation, e.g., emodin and asperthecin. 

### 3.6. CAZymes in C. protrusum

The genome of *C. protrusum* contains 412 CAZymes with a high diversity of families (Supplementary Tables S5 and S6), including 190 (46.12%) glycoside hydrolases (GH), 77 (18.69%) auxiliary activities (AA), 77 (18.69%) glycosyltransferases (GT), seven (1.70%) polysaccharide lyases (PL), 54 (13.12%) carbohydrate esterase’s (CE), and one (0.24%) carbohydrate-binding molecule (CBM). The number of CAZymes possessed by *C. protrusum* is more than that of *E. weberi* (245) and *T. reesei* (366) but is less than that of *T. virens* (484). Most of the differences between *C. protrusum* and *T. virens* can be attributed to the high copy number of GH and CE families. CAZymes involved in oxidative degradation of lignin-based components of the cell wall (10.92%, AA7 = 45) were the most abundant in *C. protrusum* followed by enzymes associated with hydrolysis of carbohydrate and non-carbohydrate substrates (CE10 8.49% (35 genes) and GH18 7.04% (29 genes), respectively). GH and GT enzymes had the largest average sets of genes among the four pathogens studied. The most abundant GH family in *C. protrusum* was GH18 7.04% (29 genes), followed by GH16 with 3.88% (16 genes) and GH3 with 3.40% (14 genes) (Figure 2C). AA13, GH43_14, GH5_11, and GT54 were found to be present in *C. protrusum* but absent in the other three pathogens studied. Other starch degrading enzymes found in all of the species compared were α-amylases of GH13, glucoamylases of GH15, and α-glucosidases of GH31. Therefore, the *C. protrusum* genome contains diverse gene families associated with fungal cell wall synthesis, modification, and degradation.

### 3.7. Secondary Metabolites in C. protrusum

The genome of *C. protrusum* was enriched with 143 SM gene clusters based on the antiSMASH database using the fungiSMASH option [64]. Only 16 gene clusters had a known function, and the remaining had largely unknown functions and were unique to the fungus (Supplementary Table S10). However, the genomes of *E. weberi*, *T. reesei*, and *T. virens* produced 35, 29, and 106 gene clusters, respectively. Many of the SM gene clusters in *C. protrusum* were grouped into 39 (27.27%) PKSs, 19 (13.29%) terpenes, 17 (11.89%) non-ribosomal peptide synthases (NRPSs), and 13 (9.10%) type 1 PKS-NRPSs (Figure 2B). The number of PKSs in *C. protrusum* was higher than in *Trichoderma* spp. *C. protrusum* has only one NRPS gene cluster (cluster 60), which encodes the apicidin biosynthetic gene cluster siderophore and seven hybrids with type 1PKS. It shares one known gene cluster with *E. weberi,* a fungal antibiotic isoflavipucine, and one with *T. reesei,* an antibiotic LL-Z1272beta, and four known gene clusters with *T. virens*, including nivalenol, vitamin B synthesis biotin, initiate apoptosis cytochalasin, and destruxins. In addition, based on the NaPDoS analysis [65], *C. protrusum*, contains 163 genes that have various functions such as antibiotic, anticancer, anti-inflammatory, immunosuppressant, and phytotoxin functions (Figure 2D). These findings suggest that *C. protrusum* has the potential to produce biologically active compounds.

### 3.8. Secretory Protein- and Pathogenicity-Related Genes of C. protrusum

**Figure 3:**
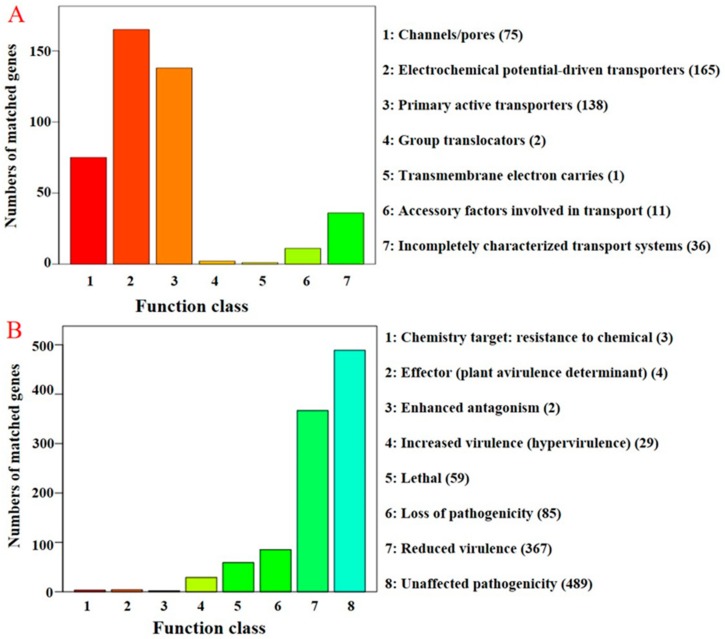
Distribution of the number of membrane transport proteins and pathogen–host interaction genes in the *C. protrusum* genome. (**A**) The distribution of membrane transport proteins (TCdb database) in *C. protrusum*; (**B**) the distribution of pathogen–host interaction (PHI) genes in *C. protrusum*. The legends on the right of each graph show the various classifications for each database used.

The genome of *C. protrusum* was predicted to encode 807 secretory proteins and 428 membrane transport proteins (TCdb database) (Figure 3A, Supplementary Tables S11 and S12). Among these secretory proteins, 378 (46.84%) were predicted to encode cell surface proteins including transmembrane proteins and glycosylphosphatidylinositol GPI-anchored proteins [57], 180 were predicted to encode proteases [59], 180 (43.69%) were predicted to encode CAZymes [63], and 180 were predicted to encode (17.34%) pathogen–host interactions (PHI) [61]. We found that only two secretory proteins were membrane transport proteins. The secretory and membrane transport proteins of *C. protrusum* are similar to the genera *Escovopsis* and *Trichoderma* [66,67]. There are much fewer proteases in *C. protrusum* (Supplementary Table S13) compared to in *Trichoderma* spp. [66], and its common proteases include aspartyl protease, serine carboxypeptidase, lipase, the peptidase family, and subtilase. We also identified 53 (0.48%), 40 (0.36%), and 184 (1.67%) genes that encode for the important family of ATP-binding cassette (ABC) transporters, MFS transporters, and cytochrome P450 (CYP) (Supplementary Table S14), respectively.

The complete protein sequences were searched against the PHI [61] and DFVF [62] databases to identify pathogenicity-related genes. We observed a total of 1038 and 453 PHI and DFVF genes, respectively (Figure 3B, Supplementary Tables S15 and S16). Moreover, 47.02% (213) of the DFVF genes were found in the PHI database. The phenotypic classification of PHI genes was classified as follows: chemistry target (0.29%, 3), effectors (plant avirulence determinant) (0.348%, 4), enhanced antagonism (0.19%, 2), increased virulence (hypervirulence) (2.79%, 29), lethal (5.68%, 59), loss of pathogenicity (8.19%, 85), reduced virulence (35.36%, 367), and unaffected pathogenicity (47.11%, 489). For example, we identified the effectors PHI:2118, PHI:2216, PHI:325, and PHI:3123 in the *C. protrusum* genome.

The *C. protrusum* genome encodes 17 (0.16%) fungal G protein coupled receptors (GPCRs); out of these, 12 share homologies with Pth11-like GPCRs (Supplementary Table S17). The number of total GPCRs and Pth11-like GPCRs in the *C. protrusum* genome is much less than in *Trichoderma* spp. from the 65 and 76 putative GPCRs encoded in the *T. atroviride* and *T. virens* genomes, respectively, but much higher than in *E. weberi* [67]. The *C. protrusum* genome contains many other genes, such as hydrophobins, *Thctf1, PacC T2* family RNases, *NPP1* (necrosis-inducing protein), GLEYA adhesin domain proteins, killer toxins, and *MD-2-related lipid-recognition* genes, which take part in host pathogenicity, pathogen–host interactions, nutrient acquisition, and adaptation to environmental stress.

Further, we explored the *C. protrusum* genome for mutations in genes that confer antifungal drug resistance by searching the Mycology Antifungal Resistance Database (MARDy) [68]. Out of the 36 antifungal drug resistance gene types in MARDy, nine different genes (*BcSdhB*, *cox10*, *cytb*, *CYP51*, *DHFR*, *DHPS*, *FKS1*, *FUR1*, and *tub2*) were found in *C. protrusum* genome (Supplementary Table S18). There were no mutations in these nine genes, which may indicate a lack of antifungal drug resistance. Hence, there is a need to perform fungicide sensitivity tests to confirm this result. 

## 4. Discussion

*C. protrusum* is a problematic pathogen that affects mushrooms. Little is currently known about its genomic sequence and structure. In the present study, we performed genome sequencing of *C. protrusum* and a comparative genome analysis to provide insights into its pathogenicity mechanisms. The *C. protrusum* genome size of 39.09 Mb is similar to other reported sizes of Hypocreaceae fungi, which range from 27.14 Mb (*Escovopsis* spp. *AC*) to 40.98 Mb (*T*. *harzianum* has the largest size in the family so far) [66,67,69]. To date, the genomes of at least 23 members of the family Hypocreaceae have been sequenced, 17 of which are from the genus *Trichoderma* and five from Escovopsis, while *C. protrusum* is the first sequenced genome in the genus *Cladobotryum*. The number of predicted protein-coding genes (11,003) of *C. protrusum* was also consistent with that of other Hypocreaceae fungi, e.g., *T. guizhouensis* (38.33 Mb, 11,255 protein-coding genes) and *T. gamsii* (37.91 Mb, 11,179 protein-coding genes) but lower than those with a similar genome size, e.g., *T. virens* (12,406 protein-coding genes) [66,69]. The number of transposable elements in *C. protrusum* is higher than that reported for members of the genus *Trichoderma*, which lack a significant repetitive DNA component in their genomes [67]. The TE content is variable in different organisms and may be used as a marker to distinguish between clonal populations of *C. protrusum* [70]. The TEs in *C. protrusum* may modify amino acids or contribute to genetic variation, thereby aiding populations to adapt successfully to changes in the environment [71,72]. Previous studies reported that the genome size, structure, and gene content are heavily influenced by natural selection, which is governed by the lifestyle and ecological niche of a species [73].

The genus *Cladobotryum* contains various species with both teleomorph (sexual) and anamorph (asexual) forms [11,74]. The sexual morph of the *Cladobotryum* is classified in a different taxon, which is known as the genus *Hypomyces* [11]. However, there is no known teleomorph for *C. protrusum*. Mating-type genes control sexual development in fungi [75,76]. We usually use the conserved domains and sequence similarities of MAT genes in fungi to identify the putative mating-type loci [77]. In this study, we found four *MAT1-2* genes in the *C. protrusum genome*, while *MAT1-1* genes were absent. The *MAT1-2* gene encodes the HMG-domain protein, which was highly conserved in comparison with *Metarhizium acridum*, *M. brunneum*, and other ascomycetes [78]. *M. acridum* also lacks the *MAT1-1* idiomorph. Pattemore, et al. [78] suggested that the lack of an observed sexual life cycle may be the result of a loss of gene function, the lack of an opposite mating-type, or merely, the inability to induce a teleomorph under laboratory conditions. Therefore, we suggest that *C. protrusum* is putatively a heterothallic species. Heterothallic fungi need a compatible strain carrying the opposite MAT idiomorph for sex to occur [79]. Therefore, in-depth population sampling is required to confirm if the *MAT1-1* mating-type occurs.

Members of the Hypocreaceae are widely known to have a mycoparasitic lifestyle [17,66,73]. The family Nectriaceae are known to be plant pathogens, while Clavicipitaceae and Ophiocordycipitaceae are insect pathogens as well as parasites of truffle fruiting bodies [73]. Therefore, we performed a phylogenomic analysis using *C. protrusum* and other nine species belonging to the Hypocreaceae, Nectriaceae, Clavicipitaceae, and Ophiocordycipitaceae families. The mimicked recent taxonomic classifications of Hypocreales, which diverged from the MRCA at 332.2 MYA, are in agreement with previous studies [67,80,81]. These results are consistent with recent phylogenetic analyses based on multiple sequence analysis for the family Hypocreaceae [48,67,82]. The results also indicate that *C. protrusum* and *Trichoderma* spp. are distantly related to each other at the family level, which is consistent with their previously assigned phylogenetic placement into different genera based on their morphological and molecular characteristics [66,74]. Mycotrophic behavior is an ancestral lifestyle in the family Hypocreaceae [73]. Different species in the various genera of the family Hypocreaceae have developed different ecological strategies [73,83]; some are aggressive and have a wide host range, like *Trichoderma* species, while others, like *Cladobotryum* spp. and *Escovopsis* spp., are not generally aggressive fungi, but they are highly selective mycoparasites with different species having different host ranges [73].

Compared with other species in the order Hypocreales, *C. protrusum* exhibited a combination of the largest expansion of gene families observed from both *Clonostachys rosea* and *Trichoderma* spp. [17,73]. These expanded gene families encode proteins related to stress, such as transporters, receptors, cell wall proteins, carbohydrate-active enzymes, and SMs (exhibiting high interspecific copy number variation), which also underwent positive selection during the evolution of *C. protrusum*, implying their importance in pathogenicity, adaptation to diverse ecological niches, and host lifestyle [84]. However, the contracted gene families in *C. protrusum* have only one known gene annotation, which is an MFS with high similarity to the one found in *Ophiocordyceps sinensis* and is known to facilitate nutrient transportation [85,86]. Therefore, the expansion of multiple gene families may play a significant role in the pathogenesis and antifungal resistance of *C. protrusum* [87].

Vegetative incompatibility or HET was observed in the *C. protrusum* genome, which is a widespread phenomenon in filamentous fungi [88]. Other proteins associated with HET are the domains of ankyrin, NACHT, and NTPase. There are 83 HET genes in the *C. protrusum* genome, which is much more than the amount observed in other fungi [88,89,90]. The HET locus inhibits the fusion between two genetically incompatible individuals by forming a fusion cell and undergoing programmed cell death. Ankyrin proteins mediate the protein–protein interactions among HET proteins [88], while NACHT domains are associated with the regulation of apoptosis/programmed cell death in fungi [90]. The lower content of TEs and the lack of a known sexual stage for *C. protrusum* might have influenced the high HET observed in the genome. Therefore, vegetative hyphal fusion controlled by HET genes may be a source of genetic variation, which is vital for the generation of the variability necessary for the adaptation to the environment and to host defense mechanisms [88].

Mycoparasitism depends on a combination of events that include lysis of the cell wall of the host. The number of CAZymes identified in the *C. protrusum* genome was similar to the average reported in other Ascomycetes fungi [91]. Among these CAZymes, the GH18 family is chitinase (-like) proteins associated with the degradation of chitin [92]. The mushroom cell wall is mostly composed of chitin; therefore, chitinolytic enzymes are key factors in mycoparasitic attack [92,93,94]. Hence, we suggest that the high number of GH18 family members in *C. protrusum* might be mostly used for mycoparasitic attack on mushrooms. The *C. protrusum* genome also includes seven genes encoding GH55 (β-1,3-exoglucanase), and the number of GH55-encoding genes is greater in mycoparasitic *Trichoderma* spp. in comparison with other filamentous fungi (28). Furthermore, most fungi have only one or two chitosanases (GH family 75), while *C. protrusum* have five, similar to the mycoparasitic *T. virens* and *C. rosea* [17,66,73]. Therefore, the results suggest that these cell wall degenerated enzymes play important roles in mycoparasitism for *C. protrusum*.

Comparative analyses of gene content, or paralogous gene number gains or losses, are extensively used to identify genes that are key determinants for ecological niche adaptation [17,66,73,95]. Here, we performed a comparative genome analysis of *C. protrusum* against *E. weberi*, *T. reesei,* and *T. virens* which belong to the same family (Hypocreaceae). *C. protrusum* and *E. weberi* were shown to share the most orthologous gene families. Previous studies showed that SMs produced in fungi are essential for defense, interaction with other organisms, and adaptation to environmental stress [73,95,96]. Cladobotryum species have been known to produce SMs (antibiotics) for years [97,98,99,100]. These were all higher compared to the other mycoparasites, with the exception of 39 putative gene clusters (cf., putative, a secondary metabolite-related protein that does not fit into any other category) [64], which was higher in *T. virens* (62). The majority of the predicted proteins were similar to proteins present in species of Sordariomycetes, and, in some cases, the best hits were found for species in the Eurotiomycetes taxa.

The PKSs and terpene synthase were shown to belong to SMs, including substances with mycotoxins, conidia, and mycelial pigmentation as well as those with antibiotic, anticancer, anti-cholesterol, anthelmintic, and insecticidal properties, and cholesterol-lowering agents. PKSs and terpene synthase were implicated in the competition and communication between microbes. As mentioned above, the number of PKSs in *C. protrusum* was shown to be higher than in *Trichoderma* spp., and this may be attributed to the higher content of mycotoxin and other genes associated with pigmentation. For instance, the red color of *C. protrusum* mycelia is due to biosynthesis of bikaverin, which was originally found in *Fusarium* species [101] and also acts as an antibiotic against different organisms, such as protozoa, oomycetes, and nematodes. The terpene gene cluster in *C*. *protrusum* encodes 4,4′-piperazine-2,5-diyldimethyl-bis-phenol, which has high homology to *Aspergillus flavus* and has possible pharmacological properties [102]. Its associated hybrids encode mycotoxins (nivalenol/deoxynivalenol/3-acetyldeoxynivalenol and trichothecene) and phytotoxins (betaenone (A, B and C)). The mycotoxins are similar to those produced by *Aspergillus nidulans*, *Fusarium* spp., and *T. virens*. In addition, we found three velvet genes in the genome. These genes are known to regulate secondary metabolism and mycoparasitism in *Trichoderma* spp. The SM clusters predicted in *C. protrusum* require complete metabolome profiling to confirm the compounds identified.

The secretory proteins are also considered to be important for virulence in fungi, such as proteases, PHI, ABC transporters, CYP, and GPCRs [17,66,93]. Several extracellular proteases including aminopeptidase, metalloproteases, serine carboxypeptidase, lipase, and subtilisin-like proteases were found to play roles in mycoparasitism in *Trichoderma* spp. [17,93,103]. The four known effectors of PHI identified in *C. protrusum* were PHI:2118, PHI:2216, and PHI:325, which were found to cause rice blast disease (*Magnaporthe oryzae*), and PHI:3123, which was found to cause anthracnose (*Colletotrichum orbiculare*) in cucurbits. Two predicted PTH11-encoding genes are also induced in *T. ophioglossoides* during growth on truffle cell wall containing media (34), emphasizing the importance of PTH11-type receptors in Hypocrealean mycoparasites. These related genes are suggested to contribute to the pathogenicity and lifestyle of *C. protrusum*.

Several authors [8,104,105,106] have reported fungicide resistance in *Cladobotryum* spp. Ma et al. [107] reported that long interspersed element (LINE) transposon of the 14α-demethylase gene (*CYP51*) confer resistance to sterol demethylation inhibitor (DMI) fungicides in *Blumeriella jaapii*. *C. protrusum* genome containing LINE (0.60%). It can therefore be inferred that fungicide resistance in *Cladobotryum* spp. may be a result of mutations in one of the target genes (*BcSdhB*, *cox10*, *cytb*, *CYP51*, *DHFR*, *DHPS*, *FKS1*, *FUR1*, and *tub2*) observed in *C. protrusum*. Currently, measures used to control cobweb disease include strict hygiene, the isolation of infected parts by covering with thick-damp paper to prevent conidial dispersion leading to further outbreaks, and application of fungicides [8,10,11]. This work suggests that it is likely that benzophenone, pyrimidinamines and quinazoline fungicides targeting actin cytoskeleton-regulatory complex protein (PF12761), and (PF12853) NADH-ubiquinone oxidoreductase, respectively, may be valuable for controlling *C. protrusum*. In addition, point mutations in ERG11 gene (*cytochrome P450 lanosterol 14α-demethylase*) which confer azole resistance in *Candida albicans* and *Cryptococcus neoformans* [108,109] were not found in the *C. protrusum* genome. Therefore, any new fungicide targeting the ergosterol biosynthesis ERG4/ERG24 family (PF01222) gene will also be useful for controlling *C. protrusum*. 

## 5. Conclusions

In this study, we sequenced the genome of *C. protrusum*, a pathogen that causes cobweb disease on cultivated edible mushrooms, using the PacBio sequencing platform. The 39.09 Mb genome with 11,003 coding genes is the first sequenced genome for the genus *Cladobotryum*. The analysis confirmed that the fungus belongs to the family Hypocreaceae, and genes from CAZymes, SMs, P450, and PHI all contribute to its mycotrophic lifestyle. Further analysis revealed that *C. protrusum* harbors arrays of genes that potentially produce bioactive SMs and stress response-related proteins that are significant for adaptation to living in hostile environments. Knowledge of the genome sequence will foster a better understanding of the biology of *C. protrusum* and mycoparasitism in general as well as aid in the development of effective disease control strategies to minimize economic losses from cobweb disease in cultivated edible mushrooms.

## Figures and Tables

**Table 1:** The genome features of *C. protrusum*.

Genome Features	*C. protrusum*
Genome size (Mb)	39.087
Total number of scaffolds	18
Total length of scaffold sequences (Assembly size)	39,087,229 bp
Scaffold N50	4,973,539 bp
Scaffold N90:	1,928,814 bp
GC-content (%):	47.84%
N Length:	0bp
N content (%):	0.0%
Transposable elements (%)	2.59
Predicted proteins	11,003
tRNA	242
rRNA	225
miRNA	97
snRNA	22

## Supplementary Materials

The following are available online at http://www.mdpi.com/2073-4425/10/2/124/s1, Tables S1–S18.
