# Supplementary material for: Genome Sequencing of Cladobotryum protrusum Provides Insights into the Evolution and Pathogenic Mechanisms of the Cobweb Disease Pathogen on Cultivated Mushroom
Source: Genes (Basel). 2019 Feb 8;10(2):124. doi: 10.3390/genes10020124 (PMC6409746; doi:10.3390/genes10020124)
Supplement: Supplementary file 1 [file genes-10-00124-s001.zip › Supplementary/genes-437115-supplementary_FigureS1-3.pdf]

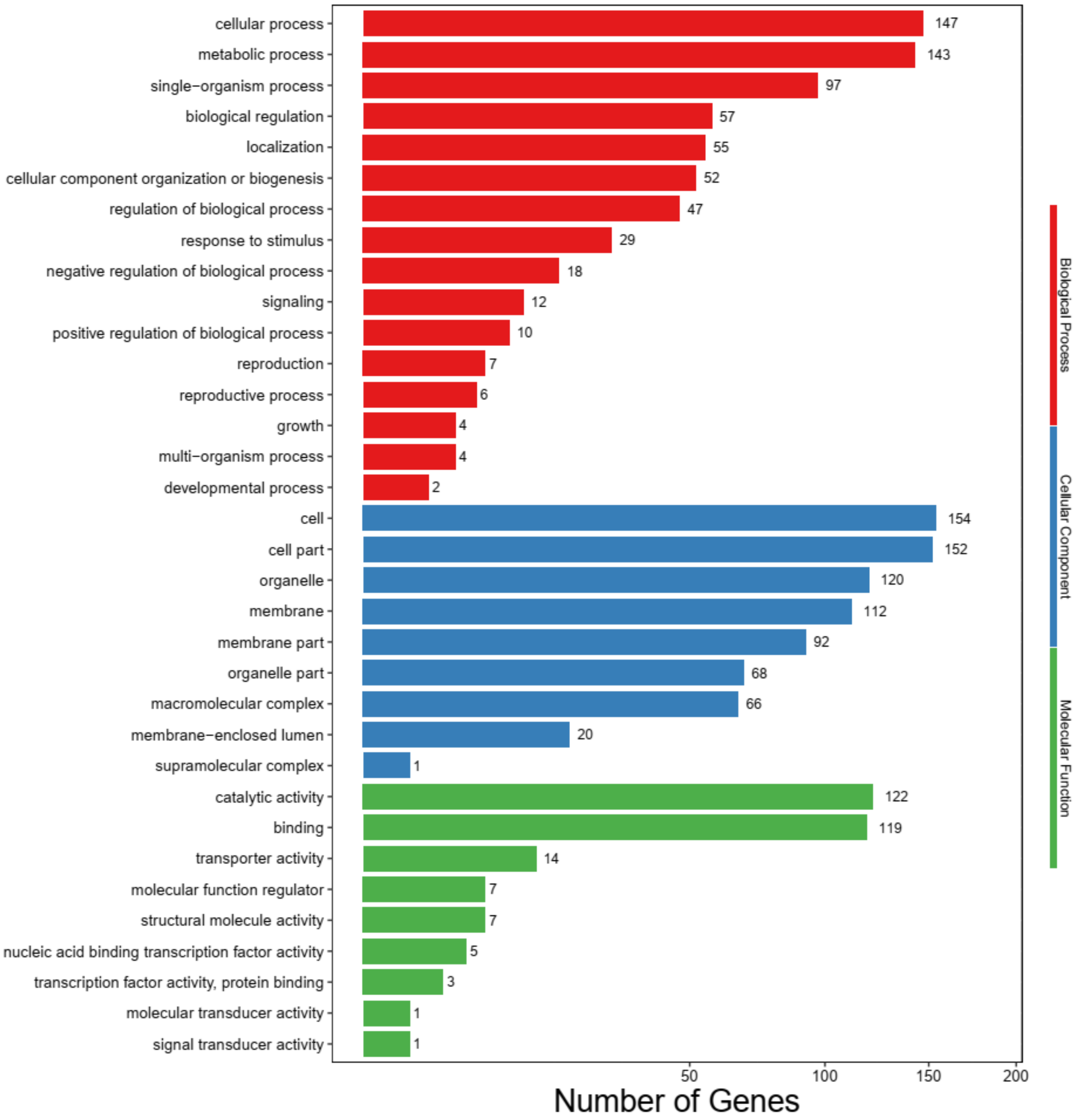

Figure S1. Gene Ontology (GO) classification of the positive selection gene in *C. protrusum*

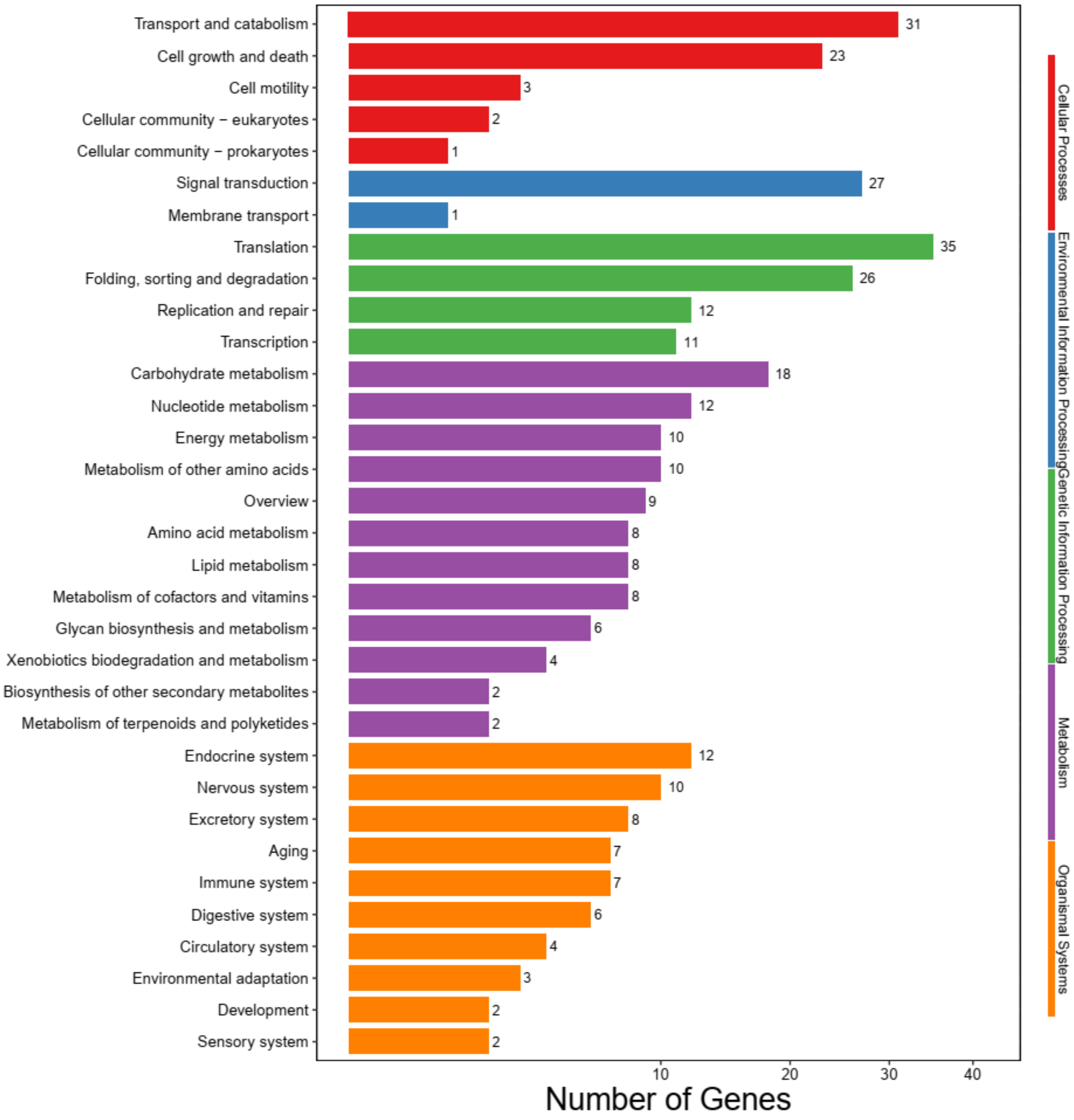

Figure S2. KEGG classification of the positive selection genes in *C. protrusum*

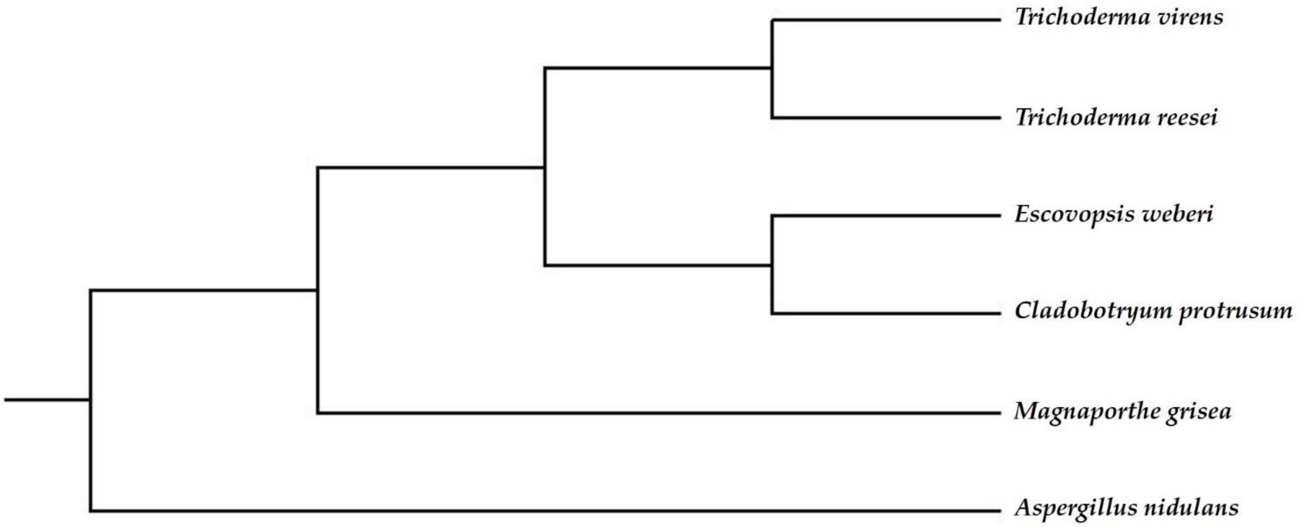

Figure S3. Phylogenetic analysis of *C. protrusum* and three fungi in the family Hypocreaceae. *Magnaporthe grisea* and *Aspergillus nidulans* are outgroups.
